# Language Learning Motivation and Burnout Among English as a Foreign Language Undergraduates: The Moderating Role of Maladaptive Emotion Regulation Strategies

**DOI:** 10.3389/fpsyg.2022.808118

**Published:** 2022-02-04

**Authors:** Xiaoxiao Yu, Yabing Wang, Fangsong Liu

**Affiliations:** ^1^School of Foreign Languages, Guangzhou City University of Technology, Guangzhou, China; ^2^Center for Linguistics and Applied Linguistics, Guangdong University of Foreign Studies, Guangzhou, China; ^3^School of English Education, Guangdong University of Foreign Studies, Guangdong, China; ^4^Department of Applied Psychology, Guangdong University of Foreign Studies, Guangdong, China

**Keywords:** burnout, emotion regulation strategies, moderation, motivation, language learning

## Abstract

In the context of English as a Foreign Language (EFL), burnout study dominantly revolves around teachers but learners’ academic burnout is largely underexplored. Academic burnout is a concerning issue worldwide that is particularly predicted by academic motivation. However, we know little about the association between motivation and burnout among EFL learners and whether maladaptive emotion regulation strategies (ERS) could moderate their association. To fill this research gap, we recruited 841 EFL undergraduates from two universities in China. Descriptive analysis showed that participants reported high levels of language learning burnout. Correlational and bootstrapped moderation analysis found that motivation and maladaptive ERS were significantly correlated with burnout in opposite directions and the correlation between motivation and burnout was significantly influenced by students’ use of two maladaptive ERS (avoiding and venting). The more frequently students chose to avoid and vent unpleasant feelings, the protective role of motivation on burnout was weaker. The implications of these findings are discussed.

## Introduction

The notion of burnout was originally conceived among staff referring to depression-like symptoms due to chronic work-related stress (Freudenberger, 1974). According to the most consensual of diversified definitions, it is a multi-dimensional syndrome comprising three symptom clusters (Maslach et al., 1997): emotional exhaustion (feelings of stress and chronic fatigue), cynicism (also known as depersonalization; a detached attitude toward work and work-related people), and lack of personal efficacy/accomplishment (reduced feelings of efficiency and success). Whilst emotional exhaustion was deemed as an emotional component, cynicism and lack of personal efficacy were regarded as cognitive ones (Maslach et al., 1997).

In educational settings, teachers’ burnout has been extensively investigated (see Kyriacou (1987), Ghanizadeh and Jahedizadeh (2015b) for reviews). With theoretical refinement and further study, however, the scope of burnout has been extended to students (Schaufeli et al., 2002; Karimi and Fallah, 2019; Madigan and Curran, 2020; Tang et al., 2021). This is because the structured activities (e.g., completing assignments, team projects) students engage resemble those at work (Schaufeli and Taris, 2005). Excessive academic demands, fear of negative evaluation from professors, overcrowded classroom, and lack of sufficient support precipitate students’ academic burnout (Pala, 2012).

Unsurprisingly, academic burnout is associated with a range of detrimental outcomes including absenteeism, dropout, academic underachievement and mental ill-being (Wang et al., 2018; Madigan and Curran, 2020). A longitudinal study found that academic burnout among university students could transit to and predict job burnout two years after graduation (Robins et al., 2018). Thus, examining protective and risky factors of academic burnout has both pedagogical and psychological implications.

In fact, a large number of antecedents of academic burnout have been examined, including external (i.e., environment-related) and internal (i.e., individual-related) ones. In terms of internal factors, maladaptive perfectionism (Zhang et al., 2007), extrinsic motivation (Chang et al., 2015), and maladaptive emotion regulation strategies (Vinter et al., 2020) were positively correlated to burnout whereas mindfulness (Yuan et al., 2018) and resilience (Ríos-Risquez et al., 2016) served as buffers against it.

Among the extant literature on academic burnout, most studies were done among students of medicine and nursing (e.g., Dunn et al., 2008; Ríos-Risquez et al., 2016). In recent years, this concept has also been increasingly investigated in the field of English as a Foreign Language (EFL). Language learners are prone to burnout due to the “psychologically unsettling” (Horwitz et al., 1991) nature of language learning process characterized by frequent classroom interactions and evaluations, as well as common anxiety and apprehension related to language input and output (Sakai and Kikuchi, 2009; Jahedizadeh et al., 2016). The contextual demands are even greater for Chinese EFL learners for whom English has been a compulsory subject since primary school (or even kindergarten in some regions) and the score of it helps determine the enrollment in middle schools and universities. For most university students, English class is still compulsory and embedded in the faculty curriculum. In such a context wherein English learning has become a required “task” with limited autonomy, students who are not interested in English learning may find it a “burden” and thereby prone to burnout (Liu et al., 2021).

Consistent with dominant paradigm in psychology, the research in EFL field also treated burnout as a tripartite multi-dimensional concept. A number of studies explored the factors EFL teacher burnout such as personality (Pishghadam and Sahebjam, 2012), perceptions of assessment (Pishghadam et al., 2014a), life-responsive language teaching perceptions (Pishghadam et al., 2014b), and cultural dimensions (Saboori and Pishghadam, 2016). Empirical studies also exist investigating the outcomes of EFL teacher burnout such as psychological reactance and spiritual intelligence (e.g., Pishghadam et al., 2021). Compared with studies on EFL teacher burnout, less evidence exists regarding EFL learner burnout. Among the limited research, an overall medium level of burnout was pervasively found among both English and non-English majors (Gao, 2012; Wang et al., 2018). However, no study examined (1) the relationship between language learning motivation and burnout among Chinese EFL undergraduates and (2) the moderating role of maladaptive emotional regulation strategies. The current research aims to fill these two research gaps. According to the Job Demand-Resource model (Demerouti et al., 2001), motivation could be regarded as personal resource whilst maladaptive emotional regulation strategies as personal demand. If would be interesting to examine whether personal demand could weaken the contribution of personal resource in the development of burnout. Finding out the relationships between burnout and the two individual factors (i.e., motivation and maladaptive emotional regulation strategies) could not only help us understand the mechanism leading to academic burnout but also prioritize future prevention and intervention programs.

### Motivation and Burnout

Academic motivation has long been viewed as an important factor in school success and adjustment. According to the Self-Determination Theory (Ryan and Deci, 2002), learning motivation denotes the willingness to learn arising from inner interest, pleasure and satisfaction (intrinsic motivation), or external factors such as pursuit of reward, avoidance of punishment (intrinsic motivation). According to the dual continuum model of motivation (Pishghadam et al., 2019), motivation could be classified into four types based on whether there was engagement and involvement: active motivation (being engaged in performing something), active demotivation (engagement as mechanical rather than mental), passive motivation (cognitive thinking without action), and passive demotivation (no cognitive activity).

The contribution of motivation to learners’ psychological well-being and academic achievement has been consistently reported (see Howard et al. (2021) for a meta-analysis). Consistent with the Self-Determination Theory, the Conservation of Resources (COR) theory (Hobfoll, 2002) contended that individuals would, in face of stress, mobilize their personal (e.g., self-efficacy) or contextual (e.g., social support) resources to withstand it. Another important theory driving this study was Job Demand-Resource Model whose basic tenet was that job and individual demands contributed to burnout whilst resources withstood it (Demerouti et al., 2001). Following this logic, motivation can be viewed as a personal resource such that those who are more motivated in learning are more resistant against study stress and thereby less prone to burnout.

Guided by the above theoretical underpinnings, the link between motivation and burnout is not hard to understand. Highly motivated learners tend to experience positive emotions (e.g., enjoyment) as opposed to emotional exhaustion, engage in learning activities with greater persistence and thereby attain greater sense of accomplishment (Dresel and Hall, 2013). In contrary, those barely motivated in learning may have affective resources (e.g., enthusiasm) depleted and become less likely expend effort in learning which in turn results in lack of feelings of accomplishment (Rubino et al., 2009). In fact, the buffering effect of motivation on burnout has been intensively established across cultures, educational stages, and disciplines (Rubino et al., 2009; Chang et al., 2015; Karimi and Fallah, 2019; Azar et al., 2020; Howard et al., 2021). However, the effect may hinge on the type of motivation examined. To be specific, extrinsic motivation and demotivation (i.e., no motivation) were found to be positively related to burnout whereas intrinsic and introjected motivation negatively related to it (Chang et al., 2015; Choi et al., 2020). Other studies found that both intrinsic and extrinsic motivation were predictive of lower burnout (Azar et al., 2020). Though a handful of studies (e.g., Rehman et al., 2020) also investigated the contribution of burnout to motivation indicating a bidirectional relationship between these two variables, literature was dominated by the motivation-burnout path.

In the context of EFL, a number of studies reported the positive relationship between demotivation and burnout (Ghanizadeh and Jahedizadeh, 2015a; Jahedizadeh et al., 2016), the negative relationship between overall motivation, intrinsic motivation and burnout (Alavi and Abbasnia, 2014; Karimi and Fallah, 2019). However, their relationship is under-explored. English as a Foreign Language is in many ways different from general educational fields and the most notable differences may lie in that (1) EFL learners face various demotivators such as low language proficiency, inappropriate teaching methods, or lack of learning facilities (Jahedizadeh et al., 2016) and (2) language learning process requires a large amount of recitation, imitation and practice, which may call for long-term and sustainable motivation in order for individuals to feel energetic and thereby continue learning. Against such a background, lowly motivated individuals are less likely to work hard at language learning activities and more likely to feel exhausted and fatigued. For Chinese EFL undergraduates, their language learning process is even more demanding. As one of the largest EFL groups, Chinese students are faced with enormous academic stress in English learning (Jin and Cortazzi, 2002). For undergraduates, English learning is compulsory and evaluated *via* nation-wide standardized tests (College English Test Band4/6), the score of which is used as a graduation or recruitment criterion (Chen and Klenowski, 2009). Thus, such a unique context necessitates the exploration of burnout and its predictors among Chinese EFL undergraduates.

### Academic Emotion Regulation Strategies as a Moderator

With the increasing acknowledgment of the pivotal role of emotions in language learning (Arnold, 2011), emotional research in foreign/second language learning abounds (Liu, 2006; Imai, 2010; Dewaele and MacIntyre, 2014; Yu et al., 2015; Boudreau et al., 2018; Dewaele and Li, 2020). According to the broaden-and-build theory (Fredrickson, 2001), positive emotions could broaden learners’ activities and strength their persistence (Pekrun et al., 2002; Zhang et al., 2020) whereas negative emotions could obstruct learners’ flexibility, engagement and language proficiency (Zhang et al., 2020). Thus, skillful management of negative emotions becomes an important individual difference influencing learners’ learning outcomes and well-being.

Conceptually, emotion regulation refers to learners’ influence on when to evoke emotions, what emotions to evoke and how to express these emotions (Gross, 1998). Based on the Modal Model (Gross, 2008), there are five categories of emotion regulation strategies (ERS): situation selection (i.e., selection of situations that could evoke certain emotions), situation modification (i.e., change of situations in order to influence emotions), attentional deployment (i.e., shift of emotions in order to influence emotions), cognitive change (i.e., change of thoughts and attitudes in order to influence emotions), and response modulation (i.e., change of physiological and behavioral response to certain emotions).

The relationship between ERS and educational outcomes is complex. Generally speaking, cognitive change, acceptance, and planning are considered adaptive strategies (Gross, 2014). In other words, those who employ more frequent use of these strategies demonstrated better well-being. In contrast, expressive suppression, avoiding situations, rumination and venting are considered maladaptive (Burić et al., 2016) and associated with poor interpersonal functioning (Gross and John, 2003), loneliness (Cook and Newins, 2021), anxiety (Malik and Perveen, 2021) and burnout (Vinter et al., 2020).

Previous studies have found the moderating role of ERS between social anxiety and loneliness (Cook and Newins, 2021), mindfulness and anxiety (Malik and Perveen, 2021), and negative feedback and academic performance (Raftery and Bizer, 2009). Borrowing, again, from the Job Demand-Resource model, maladaptive ERS could be viewed as personal demand deteriorating the level of burnout and a potential moderator influencing the relationship between personal resource (in this case motivation) and burnout. In other words, although those with lower level of academic motivation are prone to burnout, this relationship may depend on whether individuals regulate their learning-related negative emotions with maladaptive ERS. However, we know little in this regard. The current study attempts to fill these research gaps and answer the following two research questions:

1) Would EFL learners’ language learning motivation and maladaptive ERS be associated with their language learning burnout?
2) Would maladaptive ERS moderate the relationship between language learning motivation and burnout?

**FIGURE 1:**
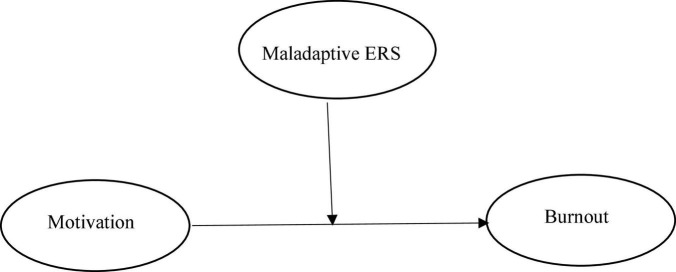
Conceptual model of the current study.

Based on the forgoing literature, we raised the following hypotheses:

1) EFL learners’ language learning motivation, maladaptive ERS would be associated with their language learning burnout;
2) The relationship between language learning motivation and burnout would be weaker for learners who use maladaptive ERS more frequently. Figure 1 shows the conceptual model of this study.

## Materials and Methods

### Participants

After obtaining ethical approval, a convenience sample of 890 non-English major undergraduates were recruited from two universities in southern China. Participants completed questionnaires online *via Wenjuanxing* (a survey platform in China, equivalent to SurveyMonkey) during College English Class after being informed of the research topic and instruction and endorsed approval of participation online. The survey was conducted on a voluntary and anonymous basis. After excluding those with massive missing data and arbitrary answers, we got a total of 841 participants (Male = 245; Female = 596). They majored in diverse disciplines including finance, communication, marketing and foreign languages other than English. Their average age was 19.57 (range 18–22). There were 427 freshmen, 393 sophomores, and 21 juniors. Seven hundred and sixteen were from University A and 125 from University B. On average, students need to attend four English classes each week (duration of each class 45 min) and spend extra 5–8 h on English after class.

### Measures

#### Academic Emotion Regulation Strategies

The Academic Emotion Regulation Strategies Questionnaire (AERQ; Burić et al., 2016) was adapted to assess participants’ language learning ERS. In this study, only the maladaptive ERS (situation avoiding, expressive suppression and venting) were assessed because they were frequently investigated and associated with burnout. The adapted questionnaire encompasses 14 items on a 5-point Likert scale (1 = strongly disagree to 5 = strongly agree). Higher scores indicated more frequent use of the target strategies. To capture ERS related to English learning, general academic context in the original scale was narrowed down into English learning context. Some items were reworded to match the Chinese EFL environment wherein all students are required to live in the dorm since the breakout of Covid-19. Therefore, statement such as “when going to school is stressful for me, I stay at home” was revised into “when English class is stressful for me, I stay at dormitory.” Confirmative factor analysis with the three subscales as indicators and maladaptive ERS as the latent construct showed good model fit (χ^2^ = 329, df = 72, RMSEA = 0.05, CFI = 0.98, TLI = 0.98, SRMA = 0.04). Cronbach’s α were 0.91, 0.97, 0.86, and 0.97 for the overall ERS, situation avoiding, expressive suppression and venting, respectively.

#### English Learning Motivation Scale

The English Learning Motivation Scale (ELMS; Peng, 2002) was used to measure participants’ English learning motivation, tapping specific types of motivation including liking and dedication on a 5-point Likert scale with higher scores indicating higher level of motivation. The original scale with 37 items was validated among Taiwanese EFL undergraduates with great psychometric properties (Pan and Wu, 2013). In this study, the item (“I don’t like English, even though I know it’s important”) was deleted due to low factor loading. Confirmative factor analysis with the five subscales as indicators and English learning motivation as the latent construct showed good model fit (χ^2^ = 2839, df = 557, RMSEA = 0.05, CFI = 0.91, TLI = 0.90, SRMA = 0.05). The Cronbach’s α were 0.97, 0.88, 0.89, 0.85, 0.86, and 0.92 for overall motivation, liking, dedication, efficacy, intrinsic, and extrinsic motivation, respectively.

#### Burnout

Students’ burnout was measured with a modified version of Maslach Burnout Inventory-Student Survey (MBI-SS; Schaufeli et al., 2002), For instance, the item “I feel emotionally drained by my studies” was rephrased in “I feel emotionally drained by my English study.” The MBI-SS is comprised of 15 items on a 7-point frequency scale (0 = never to 6 = always) with three subscales: emotional exhaustion, cynicism, and personal accomplishment (scores of this subscale would be reversed). Higher total scores indicate severer burnout. This scale has been widely used worldwide and well validated among Chinese students (Hu and Schaufeli, 2009). Confirmative factor analysis showed good model fit (χ^2^ = 393, df = 68, RMSEA = 0.08, CFI = 0.97, TLI = 0.95, SRMA = 0.04). The Cronbach’s α were 0.87, 0.95, 0.85 and 0.70 for the total scale, emotional exhaustion, cynicism and personal accomplishment, respectively. All the measures went through translation and back-translation procedure.

### Data Analysis

Confirmative factor analyses of the three variables were conducted on Mplus. A series of model-fit indices were chosen based on literature with RMSEA < 0.08, CFI > 0.90, TLI > 0.90 and SRMA < 0.08 indicating acceptable model fit (Marsh et al., 1988). SPSS 25 was used to compute descriptive statistics and correlation coefficients. If demographic variables were correlated with burnout, they would be controlled as covariates in moderation analysis. The PROCESS Macro on SPSS was used to conduct moderation analysis based on bias-corrected bootstrapped confidence intervals. This tool has been widely used to test moderation (e.g., Karababa, 2020) and was believed to be ideal owing to its robustness against abnormal distributions and inconsistent standard error equations (Hayes, 2017).

## Results

### Descriptive Statistics and Correlation

No demographic variable was correlated with the outcome (i.e., burnout). Table 1 presented the means and standard deviations of and correlations between motivation and burnout. Table 2 presented the means and standard deviations of and correlations between maladaptive ERS and burnout. The average score of burnout was 56.29 (SD = 13.45), which was higher than that reported among Iranian EFL learners (M ranging 43–45; Jahedizadeh et al., 2015, 2016).

**TABLE 1:** Correlations, means and standard deviations of motivation, burnout and subscales of them.

	1	2	3	4	5	6	7	8	9	10
1 Mot_tot	1									
2 Burn_tot	−0.61***	1								
3 Liking	0.89***	−0.60***	1							
4 Dedica	0.90***	−0.58***	0.83***	1						
5 Efficacy	0.90***	−0.61***	0.77***	0.83***	1					
6 Intrin	0.87***	−0.61***	0.82***	0.79***	0.80***	1				
7 Extrin	0.82***	−0.38***	0.59***	0.59***	0.60***	0.57***	1			
8 EE	−0.36***	0.85***	−0.37***	−0.36***	−0.39***	−0.41***	−0.17***	1		
9 Cyn	−0.40***	0.81***	−0.38***	−0.39***	−0.36***	−0.42***	−0.27***	0.68***	1	
10 RSA	−0.63***	0.63***	−0.62***	−0.59***	−0.63***	−0.57***	−0.43***	0.23***	0.21***	1
M	115.34	56.29	18.56	21.6	21.68	12.32	41.18	20.13	15.61	20.55
SD	21.59	13.45	4.28	4.8	4.87	3.32	7.57	6.44	5.23	5.96

Mot_tot: motivation; Burn_tot: burnout; Dedica: dedication; Intrin: intrinsic motivation; Extrin: extrinsic motivation; EE: emotional exhaustion; Cyn: cynicism; RPA: reduced sense of accomplishment. *** p < 0.001.

**TABLE 2:** Correlations, means and standard deviations of maladaptive emotion regulation strategies (ERS), burnout and subscales of them.

	1	2	3	4	5	6	7	8
1 Maladapitve ERS	1							
2 Burnout	0.27***	1						
3 Avoiding	0.81***	0.29***	1					
4 Suppression	0.58***	0.14***	0.19***	1				
5 Venting	0.85***	0.20***	0.62***	0.20***	1			
6 Exhaustion	0.28***	0.85***	0.25***	0.23***	0.17***	1		
7 Cynicism	0.29***	0.81***	0.31***	0.14***	0.21***	0.68***	1	
8 Reduced accomplishment	0.07	0.63***	0.10**	–0.04	0.08*	0.23***	0.21***	1
M	35.07	56.29	8.62	15.48	10.97	20.13	15.61	20.55
SD	9.21	13.45	3.79	3.67	4.73	6.44	5.23	5.96

* p < 0.05; ** p < 0.01; *** p < 0.001.

Motivation and maladaptive ERS were correlated with burnout in opposite and expected directions (*r* = −0.61, *p* < 0.001 between motivation and burnout; *r* = 0.27, *p* < 0.001) between maladaptive ERS and burnout. Specifically, liking, dedication, efficacy, intrinsic and extrinsic motivation were all negatively correlated with burnout (*r*s ranging from -0.38 to -0.61, *p* < 0.001). Avoiding, suppression and venting were all positively correlated with burnout (*r*s ranging from 0.14 to 0.29, *p* < 0.001). Focusing on the subscales, most subscales of motivation and maladaptive ERS were correlated with subscales of burnout except for suppression and reduced sense of accomplishment (*r* = 0.07, ns). Motivation and its dimensions were more correlated with reduced sense of accomplishment (*r*s ranging from -0.43 to -0.63, *p* < 0.001) as opposed to emotional exhaustion and cynicism. However, maladaptive ERS and dimensions of it were more correlated with emotional exhaustion and cynicism (*r*s ranging from 0.14 to 0.31, *p* < 0.001) as opposed to reduced sense of accomplishment.

### Moderation Models

**FIGURE 2:**
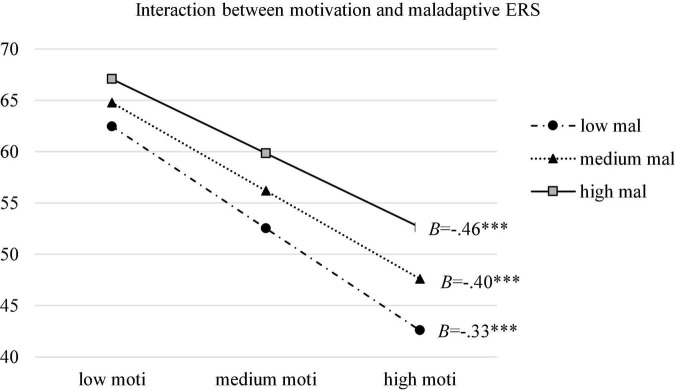
Moderation effect of overall maladaptive emotion regulation strategies (ERS) between motivation and burnout. moti: motivation; mal: maladaptive ERS; ^***^p < 0.001.

**FIGURE 3:**
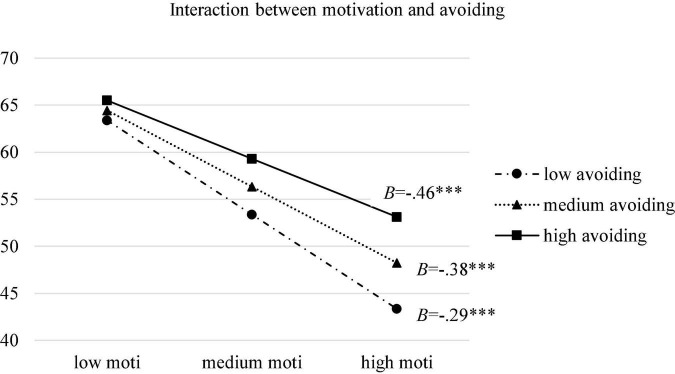
Moderation effect of avoiding between motivation and burnout. moti: motivation; ^***^p < 0.001.

**FIGURE 4:**
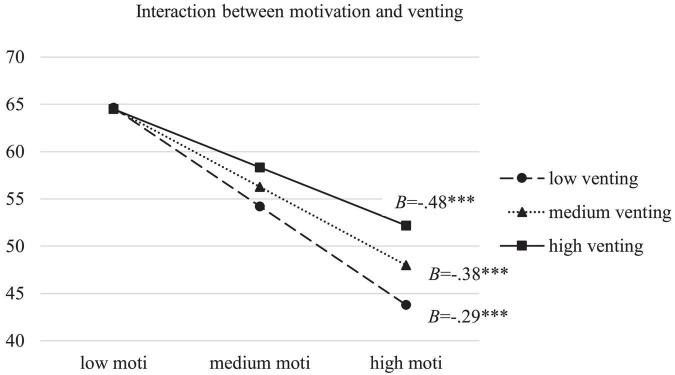
Moderation effect of venting between motivation and burnout. moti: motivation; ^***^p < 0.001.

Moderation model with overall maladaptive ERS as moderator between motivation and burnout was firstly tested. As was shown in Table 2, higher motivation was predictive of lower burnout (*B* = −0.638, SE = 0.047, 95% CI [−0.730, −0.546], *p* < 0.001). Interaction effect between motivation and maladaptive ERS was significant too (*B* = 0.007, SE = 0.001, 95% CI [0.004,0.009], *p* < 0.001), accounting for 1.8% of the variance in burnout. To find out the contribution of specific ERS, three moderation models were conducted with avoidance, suppression and venting as moderators. Results indicated that avoiding and venting, as opposed to suppression, had significant interactions with motivation in the prediction of burnout (*B* = 0.023, SE = 0.003, 95% CI [0.017,0.030], *p* < 0.001) with avoiding as the moderator; (*B* = 0.021, SE = 0.003, 95% CI [0.016,0.026], *p* < 0.001 with venting as the moderator), accounting for 3.5% and 3.9% of the variance in burnout, respectively. Simple slope tests were then carried out to examine the effect of motivation on burnout when overall maladaptive ERS and two specific significant ERS as moderators were at low (Mean–SD), medium (Mean) and high (Mean + SD) levels. Results showed that the direct effect was stronger when maladaptive ERS (*B* = −0.461, SE = 0.019, 95% CI [−0.499, −0.423] at low levels; *B* = −0.398, SE = 0.015, 95% CI [−0.428, −0.367] at medium levels; *B* = −0.334, SE = 0.019, 95% CI [−0.372, −0.297] at high levels), avoiding (*B* = −0.464, SE = 0.019, 95% CI [−0.502, −0.426] at low levels; *B* = −0.376, SE = 0.016, 95% CI [−0.406, −0.345] at medium levels; *B* = −0.287, SE = 0.020, 95% CI [−0.326, −0.249] at high levels), and venting (*B* = −0.483, SE = 0.021, 95% CI [−0.524, −0.443] at low levels; *B* = −0.384, SE = 0.016, 95% CI [−0.415, −0.353] at medium levels; *B* = −0.285, SE = 0.020, 95% CI [−0.325, −0.245] at high levels) were less frequently used. Among them, the moderation effect of avoiding was the strongest. Figures 2–4 presented the breakdown of the moderation effect of maladaptive ERS, avoiding and venting, respectively.

## Discussion

The current study aimed to find out the direct association between language learning motivation and burnout as well as the moderation effect of maladaptive ERS (a constellation of three specific ERS: avoiding, suppression, and venting) of this association among Chinese EFL undergraduates. It is the first quantitative study examining the link between language learning motivation and burnout and factors influencing this link in the Chinese EFL context. Our two hypotheses were supported with result indicating significant negative correlation between motivation and burnout and moderation effect of overall maladaptive ERS, avoiding and venting. Suppression showed no moderation effect.

### Correlational Findings

In terms of the correlation, several findings are noteworthy. Firstly, the overall negative correlation between motivation and burnout supported the Conservation of Resources model (Hobfoll, 2002), and Job Demand-Resource model (Demerouti et al., 2001) such that motivation as a personal resource served as a buffer against burnout. In other words, those who were highly motivated were less likely to experience burnout than counterparts despite similar level of study demand and stress. This finding extended the protective role of motivation to the EFL context.

Secondly, the influence of motivation and maladaptive ERS on different dimensions of burnout varied. Specifically, motivated students were notably more efficacious and, to a lesser extent, less emotional exhausted and cynical toward English learning. In contrast, students applying maladaptive ERS suffer from more emotion-related outcomes (emotional exhaustion and cynicism) and, to a less extent, reduced sense of accomplishment. In fact, there is evidence pointing to the nuanced specificity dimensions of burnout and predictors of them (Rubino et al., 2009). For example, some researchers contend that emotional exhaustion and cynicism are the core dimensions of burnout while reduced efficacy falls into the broad concept of engagement (Lee et al., 2020). From this perspective, academic maladaptive ERS was more predictive of the core elements of burnout than academic motivation. This finding is sensible and noteworthy underlining the significance of emotion regulation competence in order to combat burnout. Though we could not provide a theoretically solid explanation, this finding sheds some light on the future distinctions of burnout dimensions in research and intervention programs.

### Moderation Effect

Focusing on the moderation effect, which is the primary focus of this study, the direct effect of motivation on burnout tended to weaken with maladaptive ERS (especially avoiding and venting) more frequently used. In other words, avoiding and venting attenuated the buffering cycle from motivation to burnout. The detrimental effect of avoiding echoed avoidance-oriented and emotion-focused coping strategies (Lazarus and Folkman, 1984) which, though helpful in maintaining temporary emotional balance, was generally ineffective and associated with a range of negative outcomes (MacCann et al., 2011). Overall, the moderation effect spoke to the fact that students who deal with emotions in a maladaptive way were more susceptible to burnout even though motivation served as a buffer.

The devastating effect of venting was also consistent with previous studies. For example, one experimental study found that when participants vented anger (e.g., hitting a punching bag) they felt angrier and more aggressive than before the venting experiment (Bushman, 2002). Though some venting practices (creating a drawing depicting current mood) could shift negative emotions into positive but such effect ended after venting (Dalebroux et al., 2008). When interpreting the results of venting, it would be important to take into consideration the specific behaviors measured. In the current study, venting behaviors measured included throwing things around, yelling, and kicking, which were apparently aggressive and maladaptive. The detrimental effect of venting may be more salient for Chinese students since interpersonal harmony and interdependence is emphasized in the Chinese culture (Chan et al., 2010). Influenced by Confucianism, “forbearance” is considered a social virtue which advocates controlling of negative emotions and impulses in order to avoid interpersonal conflict. Thus, extreme emotional expression such as aggressive venting is highly disapproved and usually associated with negative outcomes.

Similarly, the moderation effect of avoiding is not hard to understand. Though inner academic motivation could boost learners’ learning efficacy and enthusiasm, this link tends to weaken if academic motivation is not translated into behavioral engagement in learning. From the perspective of psychoanalysis, emotions such as anger and fear would accumulate rather than disappear if individuals adopt avoidance-type of coping (Roth and Cohen, 1986). For Chinese EFL learners, confronting English learning problems such as fear of inaccurate pronunciation is, albeit unsettling in the beginning, beneficial for English learning buoyance in the long term.

Suppression, though correlated with burnout, failed to moderate the influence of motivation on it. Arguably, this is due to the dual-nature of suppression since previous studies were inconsistent in terms of the outcomes of it. In the academic setting, moderate suppression of negative emotions (e.g., anger) was viewed necessary or even conductive for positive emotions (Ben-Eliyahu and Linnenbrink-Garcia, 2013). It would also be interesting to investigate the effect of suppression in different cultural contexts. The maladaptive effect may become less salient in places where suppression was considered a merit and a norm (e.g., China).

The findings have several practical implications. Firstly, Chinese EFL learners are more prone to burnout than counterparts reported in non-English speaking countries such as Iran. Teachers are encouraged to attend to students’ affective factors in case the effect of learning is “filtered out”. In addition to explore the effectiveness of pedagogy, it would be necessary for teachers to screen students’ burnout level with standardized survey and adjust the complexity and acceptability of the courses when burnout is concerningly high. Though EFL teachers also reported a high level of teaching burnout (Saboori and Pishghadam, 2016), students’ burnout also deserves further attention. Secondly, given the frequent use of maladaptive emotional regulation strategies and the importance of appropriate emotion regulation, students would benefit from emotion regulation intervention programs empowering students’ ability of remediating unpleasant emotions. Even though evidence showed the effectiveness of emotion regulation intervention in reducing internalizing and externalizing problems (e.g., Houck et al., 2016), it remains unknown whether such programs could tackle burnout.

Although this study advanced our understanding about the language learning motivation and burnout as well as the role of maladaptive ERS, there are still limitations. Firstly, it is based on cross-sectional data, which precludes us from concluding cause-and-effect relationships. Even though the prediction of language learning motivation on burnout was theoretically and empirically supported, we could not rule out the possibility that burnout predicts motivation. Previous longitudinal studies found reciprocal relationship between job motivation and job burnout (ten Brummelhuis et al., 2011). It would be interesting to explore the direction of these two variables in the academic field with longitudinal data. Secondly, the data relied exclusively on self-report which may lead to bias due to social reliability tendency and memory blur. Future studies are needed with multiple sources of data, such as teacher report, peer report or experiment with emotion capture tools. Thirdly, the generalizability of results is restricted since participants were from two universities in Southern part of China. Since academic burnout is influenced by many contextual factors such as educational background and study stress, future studies are encouraged to verify the moderation model with relevant demographic information controlled.

## Conclusion

In conclusion, this study provided preliminary findings related to EFL learners’ language learning motivation and burnout. It, besides exploring the associations between these two variables, seeks to examine the moderating role of maladaptive ERS (avoiding, suppression, and venting). Bootstrapped moderation analysis found the significant moderation of avoiding and venting. Researchers are encouraged to explore when ERS moderates the association between motivation and burnout in different contexts (e.g., the most liked courses vs. the most disliked courses) and among different samples (e.g., middle school EFL learners vs. university EFL learners).

## Data Availability Statement

The raw data supporting the conclusions of this article will be made available by the authors, without undue reservation. Data would be available upon request for the corresponding author.

## Ethics Statement

The studies involving human participants were reviewed and approved by Ethical Committee of Guangdong University of Foreign Studies. The patients/participants provided their written informed consent to participate in this study.

## Author Contributions

XY and YW were responsible for the drafting, data collection, and design. FL was responsible for the data analysis and revision. All authors contributed to the article and approved the submitted version.

## Conflict of Interest

The authors declare that the research was conducted in the absence of any commercial or financial relationships that could be construed as a potential conflict of interest.

## Publisher’s Note

All claims expressed in this article are solely those of the authors and do not necessarily represent those of their affiliated organizations, or those of the publisher, the editors and the reviewers. Any product that may be evaluated in this article, or claim that may be made by its manufacturer, is not guaranteed or endorsed by the publisher.
